# Synthesis, characterization, and mechanistic insights into the enhanced anti-inflammatory activity of baicalin butyl ester via the PI3K-AKT pathway

**DOI:** 10.3389/fphar.2024.1417372

**Published:** 2024-07-22

**Authors:** Hongxu Du, Zhangxun Li, Lijuan Su, Zhengke He, Xiaoyan Tan, Fengzhi Hou, Tanjie He, Yu Pan, Shuang Xu, Liting Cao, Shiqi Dong, Yue Ma

**Affiliations:** ^1^ Department of Traditional Chinese Veterinary Medicine, College of Veterinary Medicine, Southwest University, Chongqing, China; ^2^ Immunology Research Center, Medical Research Institute, Southwest University, Chongqing, China

**Keywords:** inflammation, baicalin butyl ester, network pharmacology, molecular docking, traditional Chinese medicine

## Abstract

**Objective:**

To investigate the anti-inflammatory activity and mechanism of Baicalin derivative (Baicalin butyl ester, BE).

**Methods:**

BE was synthesized and identified using UV-Vis spectroscopy, FT-IR spectroscopy, mass spectrometry (MS) and high-performance liquid chromatography (HPLC) methods. Its anti-inflammatory potential was explored by an *in vitro* inflammation model. Network pharmacology was employed to predict the anti-inflammatory targets of BE, construct protein-protein interaction (PPI) networks, and analysis topological features and KEGG pathway enrichment. Additionally, molecular docking was conducted to evaluate the binding affinity between BE and its core targets. qRT-PCR analysis was conducted to validate the network pharmacology results. The organizational efficiency was further evaluated through octanol-water partition coefficient and transmembrane activity analysis.

**Results:**

UV-Vis, FT-IR, MS, and HPLC analyses confirmed the successfully synthesis of BE with a high purity of 93.75%. *In vitro* anti-inflammatory research showed that BE could more effectively suppress the expression of NO, COX-2, IL-6, IL-1β, and iNOS. Network pharmacology and *in vitro* experiments validated that BE’s anti-inflammatory effects was mediated through the suppression of SRC, HSP90AA1, PIK3CA, JAK2, AKT1, and NF-κB via PI3K-AKT pathway. Molecular docking results revealed that the binding affinities of BA to the core targets were lower than those of BE. The Log *p*-value of BE (1.7) was markedly higher than that of BA (−0.5). Furthermore, BE accumulated in cells at a level approximately 200 times greater than BA.

**Conclusion:**

BE exhibits stronger anti-inflammatory activity relative to BA, possibly attributed to its better lipid solubility and cellular penetration capabilities. The anti-inflammatory mechanism of BE may be mediated through the PI3K-AKT pathway.

## 1 Introduction

Inflammation is a cardinal physiological response that shields the organism against detrimental exogenous provocations. A well-regulated inflammatory reaction is instrumental for the body to counteract the invasion of pathogenic microorganisms, prevent infection propagation, purge damaged tissues, and facilitate reparative processes (Hornef et al., 2002). Conversely, dysregulated inflammatory response can precipitate a cascade of deleterious effects, ranging from tissue and organ dysfunction to multi-organ failure and fatal outcomes (Qiu et al., 2019; Zafar et al., 2021). Currently, anti-inflammatory drugs are mainly classified into two categories: corticosteroids and non-steroidal anti-inflammatory drugs (NSAIDs) (Moreira et al., 2020). Corticosteroids, such as dexamethasone, are classified as glucocorticoid, whereas NSAIDs, typified by aspirin, function as cyclooxygenase inhibitors. Although capable of alleviating the inflammatory response to a degree, both drug classes are associated with substantial side effects, posing notable drawbacks in clinical use. Glucocorticoid anti-inflammatory drugs, for instance, frequently trigger adverse effects such as osteoporosis, elevated blood glucose, and immunosuppression, while NSAIDs often cause gastrointestinal damage, liver and kidney impairment, hypersensitivity reactions, hematological anomalies, and central nervous system disorders (Rocamora-Reverte et al., 2019; Huang et al., 2022). Thus, the search for anti-inflammatory drugs with potent efficacy and minimal side effects is of imminent importance. In recent years, a growing body of research suggests that bioactive constituents within traditional Chinese medicine exhibit significant effects in modulating inflammatory responses, showcasing immense potential for development (Li et al., 2021).

Huangqin, a crucial component of traditional Chinese medicine, originates from the desiccated roots of the Labiatae plant *Scutellaria baicalensis* Georgi. According to traditional Chinese medicine theory, Huangqin is noted for its bitter flavor and cooling properties, with therapeutic effects including dampness-drying, fire-purging, detoxifying, and hemostasis actions (Gao et al., 2019). Baicalin (BA) is recognized as a primary bioactive flavonoid constituent within Huangqin. Contemporary pharmacological research has demonstrated that BA exhibits diverse biological functions, encompassing anti-inflammatory, antioxidant, antitumor, and antimicrobial activities (Liu et al., 2015). Of particular note is its remarkable anti-inflammatory activity. For instance, BA can alleviate conditions such as enteritis, hepatitis, and bronchitis through its anti-inflammatory effects. However, investigations have highlighted the suboptimal lipid solubility of BA, resulting in an oral bioavailability of merely 2.2% ± 0.2%, thereby greatly limiting its potential clinical utility (Króliczewska et al., 2017). The introduction of lipophilic fatty chains through chemical structural modification can markedly augment the compound’s lipid solubility, consequently improving both its bioactivity and bioavailability. For example, previous research reports found that Rosmarinic acid n-butyl ester exhibits a better role in countering ischemic injury to alleviate neuron damage compared to Rosmarinic acid, mediated by DAPK-p53 and suppressing microglial cell inflammatory responses (Wu et al., 2017). Furthermore, BA n-butyl ester (BE) was also found to have better intestinal protective effects compared with BA (Bao, 2022). However, the anti-inflammatory effects of BA n-butyl ester (BE) have not been reported to our knowledge. Therefore, a more convenient preparation method was firstly adopted to prepare it and the in depth mechanism was also investigated.

With the rapid advancements in disciplines such as bioinformatics, chemistry, and computer science, network pharmacology emerged by utilizing a perspective grounded in network biology to uncover the intricate interactions between drugs and biomolecules by integrating diverse sources of biological information data and following the “disease-gene-target-drug” concept of interactive networks (Hopkins, 2007; Xiang et al., 2021). Concurrently, molecular docking technology serves as a method for docking small molecules with larger molecules such as target proteins. By quantifying the interactions and binding affinities, this approach provides a targeted and innovative pathway to demystify the mechanisms of drug action (Liang et al., 2021).

Therefore, the primary objective of this study is to synthesize BE utilizing chemical modification techniques and to conduct a comprehensively exploration of its potential targets, associated signaling pathways, and biological processes using an integrated bioinformatics approach. Subsequently, we aim to establish an *in vitro* inflammatory model to validate the regulatory effects of BE on targets implicated in inflammation. This will be followed by an elucidation of its underlying mechanisms. We hold the belief that this research endeavor will not only contribute to unraveling the anti-inflammatory mechanisms of BE but also provide substantial scientific evidence for the development of novel anti-inflammatory therapeutic strategies.

## 2 Materials and methods

### 2.1 Drugs and reagents

BA (HPLC >90%) was purchased from Shanghai Aladdin Biochemical Co., Ltd. Lipopolysaccharide (LPS, from *Ecoli O55:B5*) and phosphate buffer saline (PBS) was purchased from Biosharp Life Sciences Co., Ltd. Methanol, N,N-dimethylformamide (DMF), thionyl chloride (SOCl_2_), n-butanol and dimethyl sulfoxide (DMSO) were all analytical pure reagents purchased from Shanghai Macklin Biochemical Co., Ltd. Penicillin-Streptomycin solution, Dulbecco’s Modified Eagle Medium (DMEM) and fetal bovine serum (FBS) were purchased from Gibco. PrimeScript™ RT reagent with gDNA Eraser Kit (Perfect Real Time) and TB Green^®^ Premix Ex Taq™ II (Tli RNaseH Plus) kit were purchased from TaKaRa Biotechnology Co., Ltd.

### 2.2 Synthesis of BE

**FIGURE 1:**
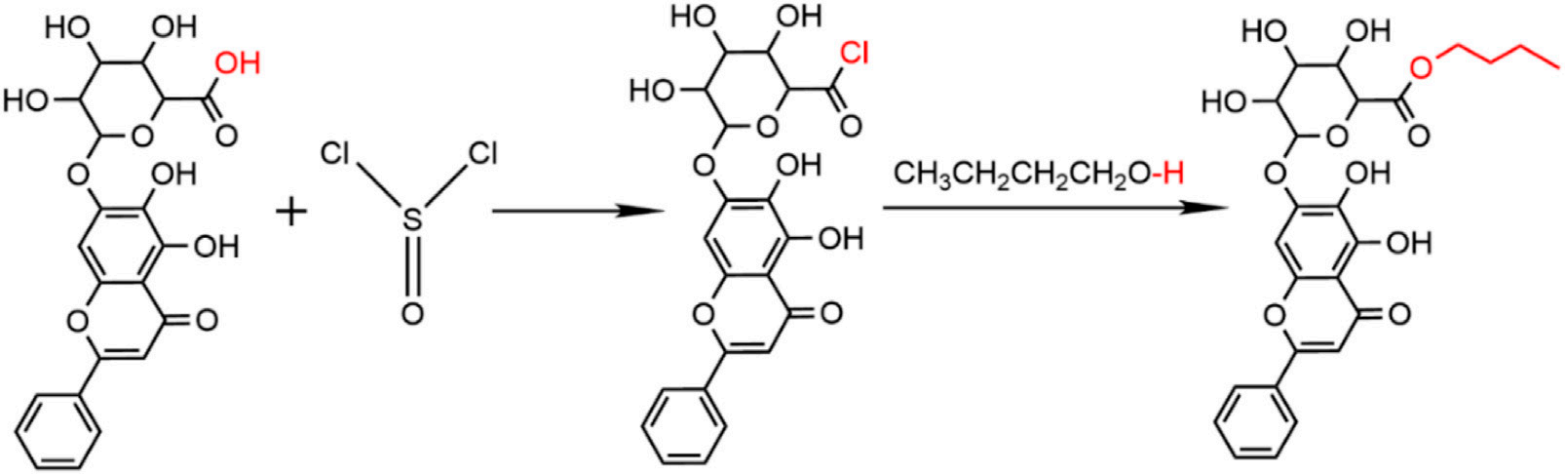
Synthesis of BE.

For the synthesis of BE, 0.3 mL of thionyl chloride (SOCl_2_) was incrementally introduced to a 10 mL n-butanol solution under ice-cooling conditions for 2 h. Subsequently, 400 mg of BA in 1 mL of N,N-dimethylformamide (DMF) was introduced slowly into the above reaction mixture stirring it at room temperature for 24 h (Figure 1). Following completion of the reaction, 0.5 mL of DMF was added to facilitate dissolution of the reaction product, which was then precipitated by slowly adding 50 mL of distilled water. The product was finally purified through repeated washing and filtration with 5 L of distilled water, and the purified solid was collected after freeze-drying with a Lyophilizer (Ningbo Xinzhi, China).

### 2.3 UV-visible spectrophotometric analysis

BA and BE were fully dissolved in methanol at a concentration of 50 μg/mL and subsequently scanned in the wavelength range of 800–200 nm using a UV-Vis spectrometer (Nanjing Feller, China). Meanwhile, the solvent of methanol was used as the blank reference solution.

### 2.4 FT-IR spectral analysis

Completely dried BA and BE powders were respectively mixed with potassium bromide KBr in a ratio of 1:100 and then ground and pressed to a thickness of less than 0.5 mm using a Nicolet iS50 FT-IR spectrometer equipped with OMNIC application software (Thermo Electron, USA). Finally the FT-IR spectra results were recorded in the wavelength range of 4,000–500 cm^−1^.

### 2.5 Mass spectrometry (MS) analysis

To investigate the molecular weight of BE, the electrospray ionization tandem mass spectrometry (XEVO TQ-S Micro, Waters Corporation, United States) was detected in positive ion mode using an electrospray ionization source (ESI) with a scan range of 100–1,000 m/z.

### 2.6 High-performance liquid chromatography (HPLC) analysis

A HPLC system (LC-20A, Shimadzu Corporation, Japan) was utilized for the detection of BA and BE. Precisely, 5 mg of BA standard and BE powder were weighed in a 10 mL volumetric flask. Afterward, they were dissolved in methanol and adjusted to 0.25 mg/mL. The resulting solution was then filtered through a 0.22 μm nylon 66 organic phase microporous filter. The binary gradient high-pressure elution method was employed using an Agilent C_18_ column (4.6 × 250 mm, 5 μm) with a mobile phase consisting of methanol, water, and phosphoric acid (47:53:0.2). The detection wavelength was set at 280 nm, the column temperature was set at 40°C, and the injection volume was set at 10 μL. The duration of all detection was set to 125 min.

### 2.7 Effect of BE on the viability of RAW264.7 cells

RAW264.7 cells were cultured in a medium composed of 10% fetal bovine serum (FBS), 1% penicillin-streptomycin, and 89% DMEM within a 5% CO_2_ incubator at a temperature of 37°C. Subsequently, cells maintained at a concentration of 3 × 10^6^ cells/mL, were subjected to varying concentrations of BA or BE (ranging from 0 to 200 μmol/L) for a duration of 24 h. Post-incubation, the culture medium was carefully removed, and each well received 65 μL of MTT (2 mg/mL), which was then left to incubate for 4 h within the controlled environment of the incubator. Thereafter, 100 μL of DMSO was introduced and gently agitated for 5 min to ensure complete dissolution of the crystalline substance. The absorbance of each well was measured at 490 nm using an Microplate reader (M1000 pro, TECAN, Switzerland) to assess the effects of various concentrations of BA and BE treatments on cell viability.

### 2.8 Anti-inflammatory effects analysis *in vitro*

The RAW264.7 cells were seeded at a density of 3 × 10^6^ cells/mL in a 48-well plate and incubated at 37°C in 5% CO_2_ for 24 h. Subsequently, the cells were divided into four groups: Cell control (CC) group, LPS group, BA group, and BE group. In the LPS group, cells were treated with 1 μg/mL of LPS for 20 h (Cho et al., 2014; Wang et al., 2018). For the BA and BE groups, cells were treated with 1 μg/mL LPS along with BA or BE for 20 h. The CC group cells cultured in regular growth medium for the same duration. Finally, cell culture supernatant was collected for NO content assessment, and cells were harvested for subsequent measurement of pro-inflammatory cytokine (IL-1β, IL-6, COX-2, and iNOS) levels. In brief, total RNA was collected, lysed, and extracted from cells of each group, the integrity of RNA was assessed, and its concentration was determined. Meanwhile, the reverse transcription reaction was meticulously conducted using the PrimeScript™ RT reagent Kit with gDNA Eraser (Perfect Real Time) in strict accordance with the instructions. Subsequently, PCR amplification was conducted using the QuantStudioTM 3 real-time fluorescence quantitative PCR system (Applied Biosystems, Thermo Fisher, USA). The PCR reaction procedures were as follows: 95°C for 30 s; followed by 40 cycles of 95°C for 5 s, 60°C for 30 s, and 95°C for 15 s; and a final extension at 95°C for 15 s, 60°C for 60 s, and 95°C for 15 s. β-actin was used as an internal reference gene, and the primer sequences are shown in Table 1.

**TABLE 1:** Primer sequences for real-time PCR.

Gene	Primer (5′–3′)	Length (bp)
IL-1β IL-6	Forward: GAAATGCCACCTTTTGACAGTG Reverse: TGGATGCTCTCATCAGGACAG Forward: AGCCTCTTCCGTGTTTCTGT Reverse: ATCTTGGAGCGAGTTGTGGATTGTC	116 121
COX-2	Forward: CATGAGCCGTCCCCTCACTAGG Reverse: AATCCTGGTCGGTTTGATGCTACTG	88
iNOS	Forward: GGAGCGAGTTGTGGATTGTC Reverse: TAGGTGAGGGCTTGGCTGAGTG	131
JAK2	Forward: GTGTGGAGATGTGCCGCTATGAC Reverse: AGTCTCGGAGGTGCTCTTCAGTG	99
PIK3CA	Forward: GCACAAGAGTACACCAAGACCAGAG Reverse: GCATTCCAGAGCCAAGCATCATTG	130
AKT-1	Forward: TCA​GGA​TGT​GGA​TCA​GCG​AGA​GTC	108
	Reverse: AGG​CAG​CGG​ATG​ATA​AAG​GTG​TTG	
NF-κB	Forward: AAA​TGG​GAA​ACC​GTA​TGA​GCC​TGT	92
	Reverse: GTT​GTA​GCC​TCG​TGT​CTT​CTG​TCA​G	
SRC HSP90AA1	Forward: TCACCGCCTCACTACCGTATGTC Reverse: CATCCACACCTCTCCGAAGCAAC Forward: ACGAAGCATAACGACGATGAGCAG Reverse: CATTGGTTCACCTGTGTCAGTCCTC	136 87
β-actin	Forward: AGAGGGAAATCGTGCGTGAC Reverse: CAATAGTGATGACCTGGCCGT	138

### 2.9 Network pharmacology analysis

#### 2.9.1 Prediction of BA and BE related targets

For the identification of potential targets for BA and BE, their structures in MOL_2_ format were independently submitted to the Pharm Mapper database (www.lib-requesting) and Swiss Target Prediction database (http://www.swisstargetprediction.ch/) to predict their potential targets. Concurrently, to comprehensively analyze the potential targets of BA, literature reporting BA targets was searched in the NCBI database (http://www.ncbi.nlm.nih.gov/pubmed/) using “baicalin” as the keyword. After combining targets from literature and databases and eliminating duplicates, the targets of BA or BE were preliminary determined. Meanwhile, considering of the shortage of the target proteins of BE predicted by the databases and the highly similar of the structure with BA, the final target proteins of BE was obtained by merging the targets of BA and BE described above.

#### 2.9.2 Inflammation-related targets prediction

In order to obtain targets for inflammatory diseases, searches were performed in the Gene Cards (http://www.genecards.org), Comparative Toxicogenomics Database (http://ctdbase.org/), DisGENT (http://www.disgenet.org), and the Online Mendelian Inheritance in Man (OMIM) (https://omim.org/) databases using the search term “inflammation.” Subsequently, the search results were merged, and duplicate targets were removed.

#### 2.9.3 Protein-protein interaction analysis

Comparisons were made between the respective target proteins of BA and BE with the targets related to inflammation. Following this, the intersected target proteins were fed into the STRING database (https://cn.string-db.org/), with a stringent confidence threshold at 0.9. Within the network, protein-protein interaction (PPI) were established, omitting disconnected or weakly connected proteins (Szklarczyk et al., 2020). The outcome was an intricate PPI network indicative of potential anti-inflammatory protein interactions. The resultant data was retrieved and subsequently imported into Cytoscape 3.9.0 for visual refinement in TSV format. By utilizing the CytoNCA plugin within Cytoscape, an extensive topological analysis of the PPI network was conducted, encompassing the computation of node degrees. This quantitative assessment paved the way for the construction of a circular protein interaction diagram, where pivotal nodes were selectively identified based on their degrees.

#### 2.9.4 Kyoto encyclopedia of genes and genomes (KEGG) pathway analysis

To explore the anti-inflammatory mechanisms of BA and BE, the core target proteins obtained were analyzed through KEGG enrichment analysis using the Metascape database (https://metascape.org/gp/index.html) and visualized by the Bioinformatics (http://www.bioinformatics.com.cn/). In brief, the target proteins with degree value ≥10 were chosen and input into the Metascape database for enrichment analysis. The threshold of the relevant pathways were defined as *p* < 0.01.

### 2.10 Molecular docking

The 3D structures of BA and BE were generated using Chem3D 21.0 software and saved in MOL_2_ format. The key protein targets of BE selected from the PPI network were obtained from the RCSB PDB database (https://www.rcsb.org/) to obtain their 3D structures. The PDB codes for the core targets utilized were as follows: SRC (4HXJ), TP53 (2K8F), IL6 (1ALU), STAT3 (6NJS), AKT1 (6HHI), TNF (2AZ5), HSP90AA1 (3WQ9), MAPK1 (6SLG), CASP3 (1NME), EGFR (5WB7), MAPK14 (3LFF), TLR2 (6NIG), NFKB1 (1IKN), TLR4 (2Z65), IL10 (2H24), PIK3CA (2V1Y), MAPK8 (4G1W), MAPK10 (3TTJ), RHOA (3LW8), and IL4 (3QB7). Autodock Tools was utilized to prepare input files for the receptor and ligands. PyMOL software was employed for visual analysis of the molecular docking results (Zhang and Sanner, 2019; Eberhardt et al., 2021).

### 2.11 Anti-inflammatory mechanism validation *in vitro*

To substantiate the molecular mechanisms predicted by network pharmacology, the influence of BE on relevant signaling pathways was explored using the LPS-induced RAW264.7 cell inflammation model. In brief, RAW264.7 cells were cultured and grouped according to the methods described in Section 2.8, total RNA was extracted, and reverse transcription reactions were performed to obtain cDNA. Finally, real-time fluorescence quantitative PCR (qRT-PCR) using the Quant StudioTM 3 system (Thermo Scientific, United States) was employed to assess the relative expression levels of target genes. Detailed primer information is provided in Table 1.

### 2.12 Oil-water partition coefficient detection

The Oil-water partition coefficient of BA and BE in n-octanol and water were determined by the shake flask-HPLC method (Brooke et al., 1990; Brusač et al., 2019). Briefly, BA and BE were individually weighed and dissolved in 2 mL of water-saturated n-octanol. After centrifugation at 12,000 r/min for 15 min, 0.5 mL of the supernatant was mixed with an equal volume of water-saturated n-octanol and incubated in a constant temperature shaking incubator (37°C, 180 r/min) for 24 h. After shaking, the mixture was allowed to stand overnight, and then the n-octanol phase and the aqueous phase were separated. Both phases were diluted tenfold with methanol, filtered through a 0.22 μm microporous membrane, and subjected to HPLC analysis to measure the peak areas. The detection conditions were as follows: UV detection wavelength of 280 nm; the mobile phase consisted of methanol-water-phosphoric acid solution (47:53:0.2); flow rate at 1.0 mL/min; injection volume of 20 μL.

The calculation formula for the oil-water partition coefficient is *P* = Co/Cw; log *P* = log(Co/Cw). Co: the concentration of the compound in n-octanol, Cw: the concentration of the compound in aqueous phase (Ebada et al., 2022).

### 2.13 Transmembrane ability detection

RAW264.7 cells were seeded in 6-well plates at a density of 3.0 × 10^6^ cells/mL and cultured at 37°C and 5% CO_2_ in a CO_2_ incubator for 24 h. After washed twice with PBS, the cells were divided into CC group, BA group, and BE group. The BA and BE groups were treated with basic DMEM medium containing 50 μmol/L of BA and BE, respectively, while the CC group received the same volume of basic DMEM medium. After 4 h treatment, the medium was removed, and cells were washed three times with PBS. Then, 1 mL of methanol was added to collect the cells, followed by 15 min of sonication to lyse the cells. The supernatant was collected after centrifugation and filtered through a 0.22 μm microporous membrane. HPLC was used to determine the content of each component, and the HPLC detection method was same as part “2.6”.

### 2.14 Statistical analysis

Statistical analysis was conducted using SPSS version 21.0. One-way analysis of variance (ANOVA) was employed to assess the significance of comparisons between groups after confirming homogeneity of variance and followed by Tukey’s *post hoc* test to determine statistical differences between groups. Statistically significant differences were defined when the *p*-value was less than 0.05. All data are presented as mean ± standard deviation.

## 3 Results

### 3.1 The characterization of BE

**FIGURE 2:**
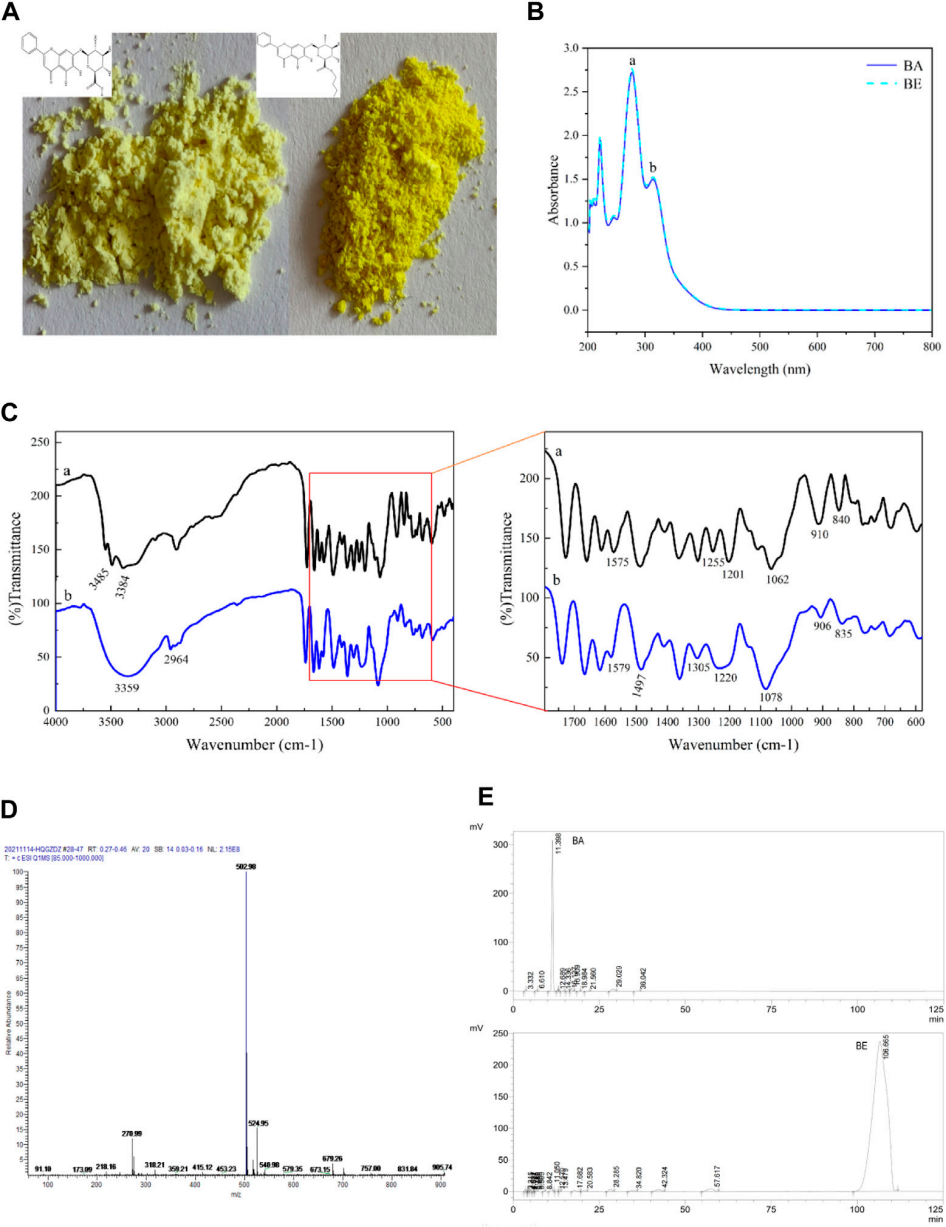
The characterization of BE. **(A)** Apparent property of BA and BE. **(B)** Full wavelength scanning of BA and BE. **(C)** FT-IR spectrum analysis of BA and BE. **(D)** Mass spectrometry detection of BE. **(E)** HPLC chromatograms of BA and BE.

As shown in Figure 2A, both BA and BE were yellow in color. BA appeared as a light-yellow powder, while BE was in the form of yellow fine particles, with a color deeper than that of BA.

BA and BE were subjected to full wavelength scanning using UV-Visible spectrophotometry, and the results are shown in Figure 2B. Both BA and BE exhibited two strong absorption peaks in the UV spectrum range of 200–400 nm, with absorption peak a at a wavelength of 278 nm and absorption peak b at a wavelength of 314 nm. The position and height of the absorption peaks of BE were consistent with those of BA, and this characteristic peak is consistent with the characteristic peak for identifying BA (Liang et al., 2009), indicating that compound BE is a derivative structurally similar to BA.

Figure 2C shows the FT-IR analysis results of BA (black) and BE (blue). Overall, the FT-IR spectrum of BA and BE exhibited remarkable similarities, although there were still certain distinctions. In Figure 2Ca, the bands at 3,485 cm^−1^ and 3,384 cm^−1^ corresponded respectively to the intermolecular hydroxyl binding peak and the free hydroxyl characteristic peak of BA molecules. The range of 1,600–1700 cm^−1^ represented the C=C and benzene ring C-H stretching vibration characteristic peaks within BA molecules. The band at 1,255 cm^−1^ corresponded to the C-O vibration in carboxylic acid, while the band at 1,201 cm^−1^ corresponded to the C-OH vibration in carboxylic acid. The appearance of these two vibration peaks could be taken as evidence of the presence of carboxylic acid functional groups in the substance. Furthermore, the vibrational absorption peak at 1,062 cm^−1^ reflected the existence of the C-O-C structure within the molecule. In Figure 2Cb, the broad peak at 3,359 cm^−1^ corresponded to intermolecular coupling resulting from the multiple free hydroxyl molecular associations of BE. Some new peaks appeared at 2,964 cm^−1^, potentially due to -CH_3_ vibration and -CH_2_ symmetric stretching vibration. Compared to the infrared spectrum of BA, the vibrational absorption peaks at 1,255 cm^−1^ and 1,201 cm^−1^ disappeared in the infrared spectrum of BE, while a new vibrational absorption peak emerged at 1,220 cm^−1^. This indicates a change in the carboxylic acid functional group in BA, leading to the formation of a new group. Meanwhile, at position of 1,078 cm^−1^, a broad peak with a relatively strong intensity and larger area appeared, may corresponded to the C-O bond of the ester.

BE was analyzed through MS detection, and its mass spectrum was recorded. As shown in Figure 2D, in the positive ion mode, the primary molecular ion peak ([M + H]^+^) was observed at m/z of 502.98, with a relative abundance close to 100%, indicating that the molecular weight of the sample’s protonated molecular ion is 502.98, which is consistent with the molecular weight result of BE (502.47) predicted by the SwissADME.

Figure 2E displays the HPLC detection results of the BA standard and BE. The characteristic chromatographic peak of BA appeared at a retention time of 11.398 min in the HPLC chromatogram of BA standard. However, no chromatographic peak of BA was detected in the HPLC chromatogram of BE. Instead, a unique and strong chromatographic peak was detected at a retention time of 106.665 min, indicating that BE exhibits stronger hydrophobicity and higher purity compared to BA. Subsequently, the purity of BE was calculated to be 93.75% using the peak area normalization method (Li et al., 2017).

### 3.2 Anti-inflammatory analysis *in vitro*

**FIGURE 3:**
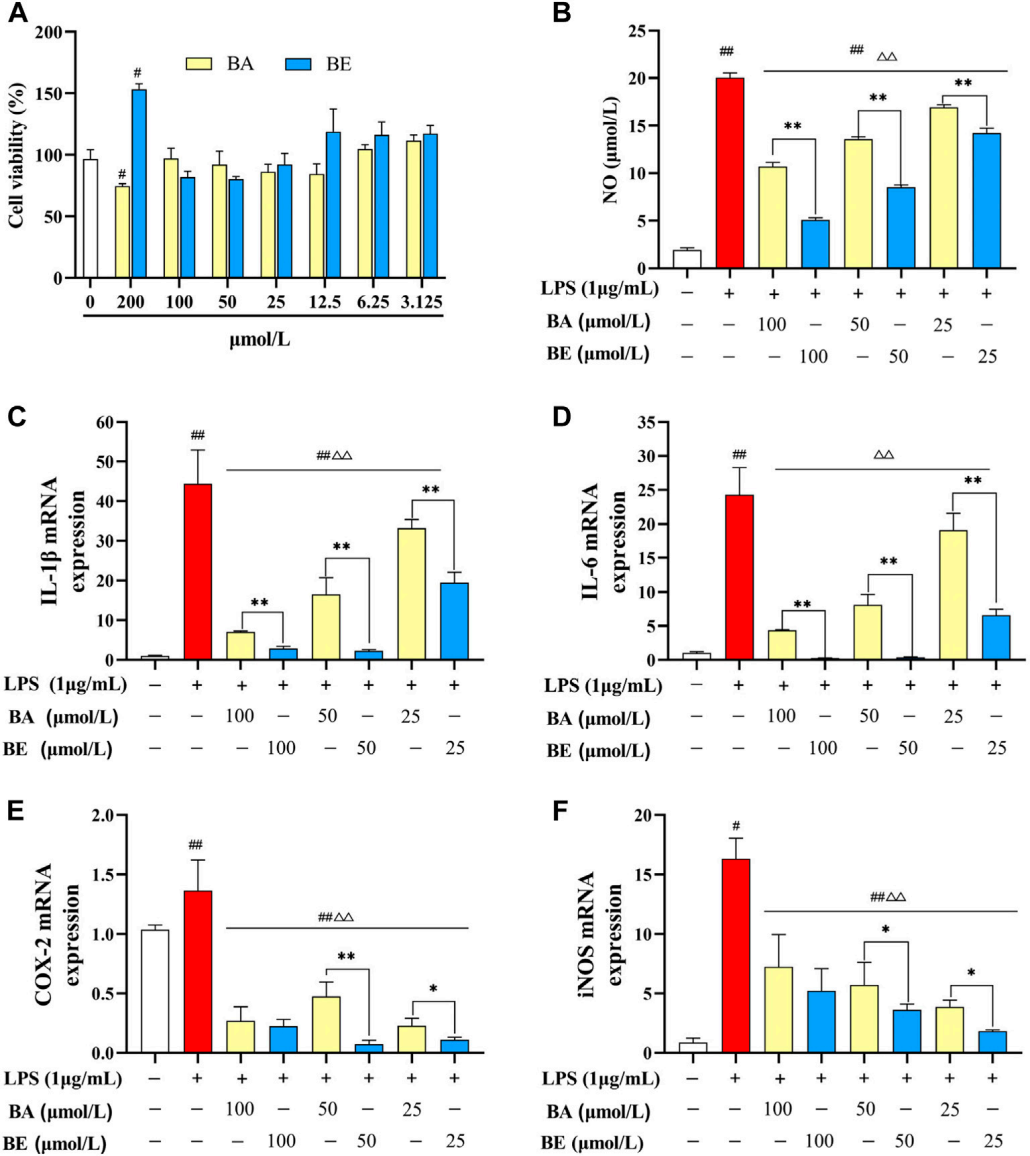
Anti-inflammatory effect of BA and BE in RAW264.7 cells. **(A)** Cell viability. **(B)** NO. **(C)** IL-1β. **(D)** IL-6. **(E)** COX-2. **(F)** iNOS. Note: #*p* < 0.05, ##*p* < 0.01 compared with the CC group; △ *p* < 0.05, △△ *p* < 0.01 compared with the LPS group; *p < 0.05, **p < 0.01 compared with BA group (N = 3).

As depicted in Figure 3A, the cell viability of RAW264.7 cells after 24 h exposure to varying concentrations of BA and BE is illustrated. Within the concentration range of 3.125–100 μmol/L, no significant differences in cellular viability were observed between the RAW264.7 cells treated with BA and BE compared to the CC group (*p* ≥ 0.05). This suggests that both compounds within this concentration range have no impact on cellular vitality. However, at a concentration of 200 μmol/L, BE exhibited a substantial enhancement in the cellular viability of RAW264.7 cells (*p* < 0.05). In light of these results, BA and BE were selected at concentrations of 100, 50, and 25 μmol/L for subsequent investigations.

The influence of multiple concentrations of BA and its derivative on the release of NO in LPS-induced RAW264.7 cells is elucidated in Figure 3B. In comparison to the CC group, the induction of NO release in RAW264.7 cells by LPS (1 μg/mL) exhibited a highly significant increase (*p* < 0.01). Following treatment with BA and its derivative at concentrations of 100 μmol/L, 50 μmol/L, and 25 μmol/L, the release of NO exhibited a markedly significant reduction in comparison to the LPS group (*p* < 0.01), displaying a dose-dependent pattern. A noteworthy observation is that, in comparison to the BA group, within the scope of experimental concentrations, the release of NO in cells treated with various concentrations of BE was markedly lower than their respective counterparts in the BA group (*p* < 0.01).

The impact of BA and its derivative on mRNA expression levels of pro-inflammatory cytokines in LPS-induced RAW264.7 cells are illustrated in Figures 3C–F. Comparatively, LPS treatment significantly elevated the mRNA expression levels of IL-1β, IL-6, COX-2, and iNOS in RAW264.7 cells relative to the CC group (*p* < 0.01). Conversely, both BA and BE treatments led to a considerable reduction in the mRNA expression levels of these pro-inflammatory cytokines in LPS-treated cells (*p* < 0.01). Notably, within the concentration range of 25–100 μmol/L, the expression levels of these pro-inflammatory cytokines were markedly lower in the BE group compared to the BA group. These findings collectively indicate that both BA and BE possess significant capacity to suppress the expression of pro-inflammatory cytokines and alleviate inflammation, with BE exhibiting a notably superior anti-inflammatory effect over BA.

### 3.3 Network pharmacology analysis

**FIGURE 4:**
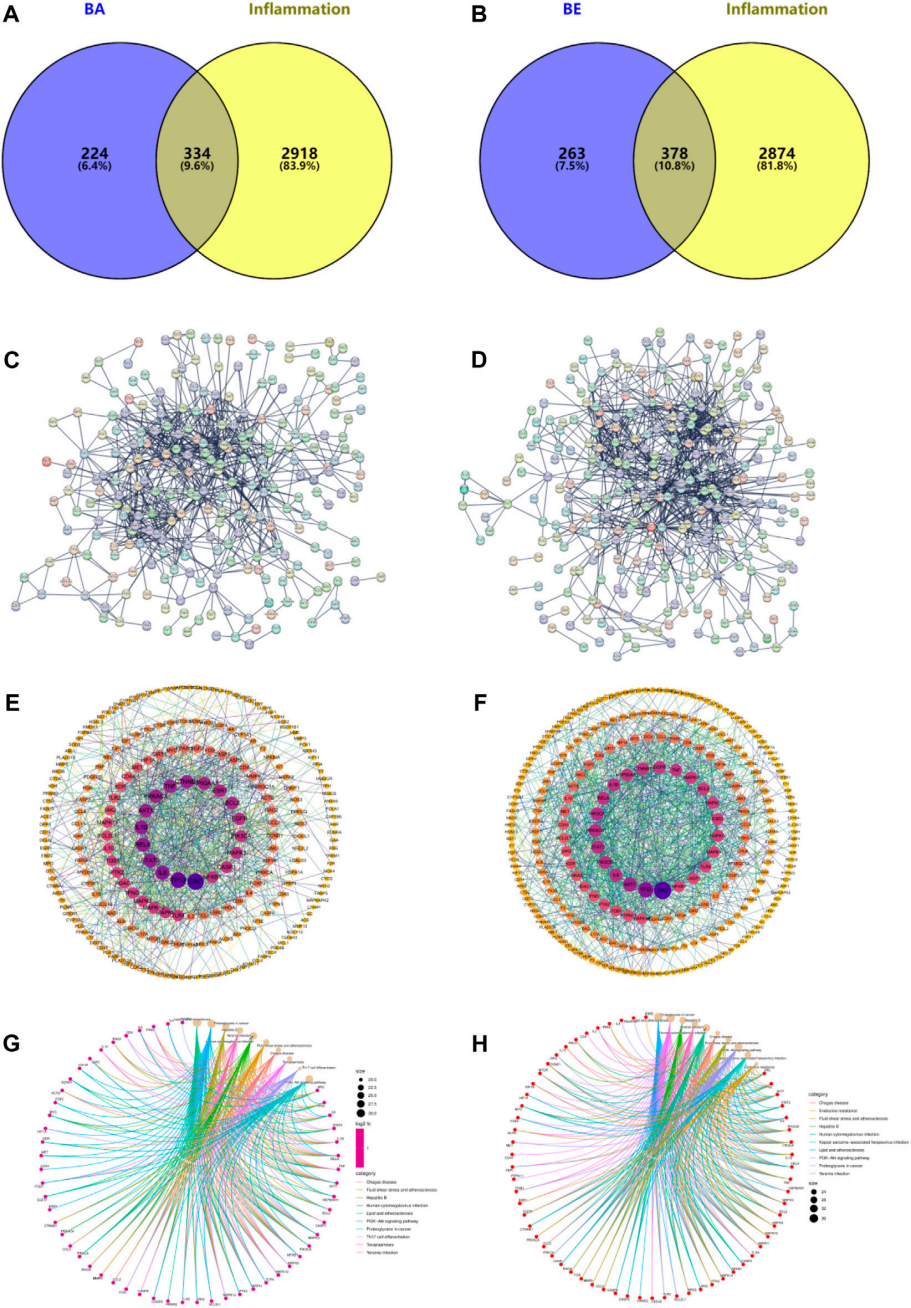
Network Pharmacology analysis. **(A)** BA intersects with inflammatory targets. **(B)** BE intersects with inflammatory targets. **(C)** Intersect targets PPI diagram of BA. **(D)** Intersect targets PPI diagram of BE. **(E)** Topology analysis of BA’s PPI network. **(F)** Topology analysis of BE’s PPI network. **(G)** The KEGG signaling pathways of BA. **(H)** The KEGG signaling pathways of BE.

In order to identify potential targets contributing to the anti-inflammatory effects of BA and BE, and to elucidate the underlying mechanisms of their actions, a network pharmacology analysis was conducted. The results are depicted in Figure 4. Through the databases such as NCBI, Gene Cards, Pharm Mapper, and Swiss Target Prediction, a total of 558 target proteins were identified for BA, and 641 potential targets were identified for BE. Based on information from Gene Cards, DisGeNET, and OMIM databases, a total of 3,252 inflammation-related target proteins were predicted. By comparing the drug target proteins with the 3,252 inflammation-related target proteins, 334 and 378 overlapping targets were identified respectively (Figures 4A,B). Thereafter, a protein-protein interaction (PPI) network of the aforementioned targets was constructed by utilizing the STRING database with the highest confidence threshold (confidence >0.9) (Figures 4C,D). Finally, topological analysis of the PPI network was conducted using the CytoNCA plugin within the Cytoscape software, resulting in the identification of 59 and 66 core target proteins for BA and BE against inflammation respectively (Figures 4E,F).

To further investigate the anti-inflammatory mechanism of BA and BE, the above 59 and 66 core target proteins were submitted to the Metascape database to perform KEGG analysis. As shown in Figure 4G, the KEGG signaling pathways of BA mainly involved the pathways of Lipid and atherosclerosis, Proteoglycans in cancer, Hepatitis B, *Yersinia* infection, Human cytomegalovirus infection, Fluid shear stress and atherosclerosis, Chagas disease, Toxoplasmosis, Th17 cell differentiation, and PI3K-AKT signaling pathway. Meanwhile, the KEGG signaling pathways of BE primarily involved pathways of Lipid and atherosclerosis, Proteoglycans in cancer, Hepatitis B, *Yersinia* infection, Human cytomegalovirus infection, Chagas disease, Fluid shear stress and atherosclerosis, PI3K-AKT signaling pathway, and Kaposi sarcoma-associated herpesvirus infection (Figure 4H).

### 3.4 Molecular docking

**FIGURE 5:**
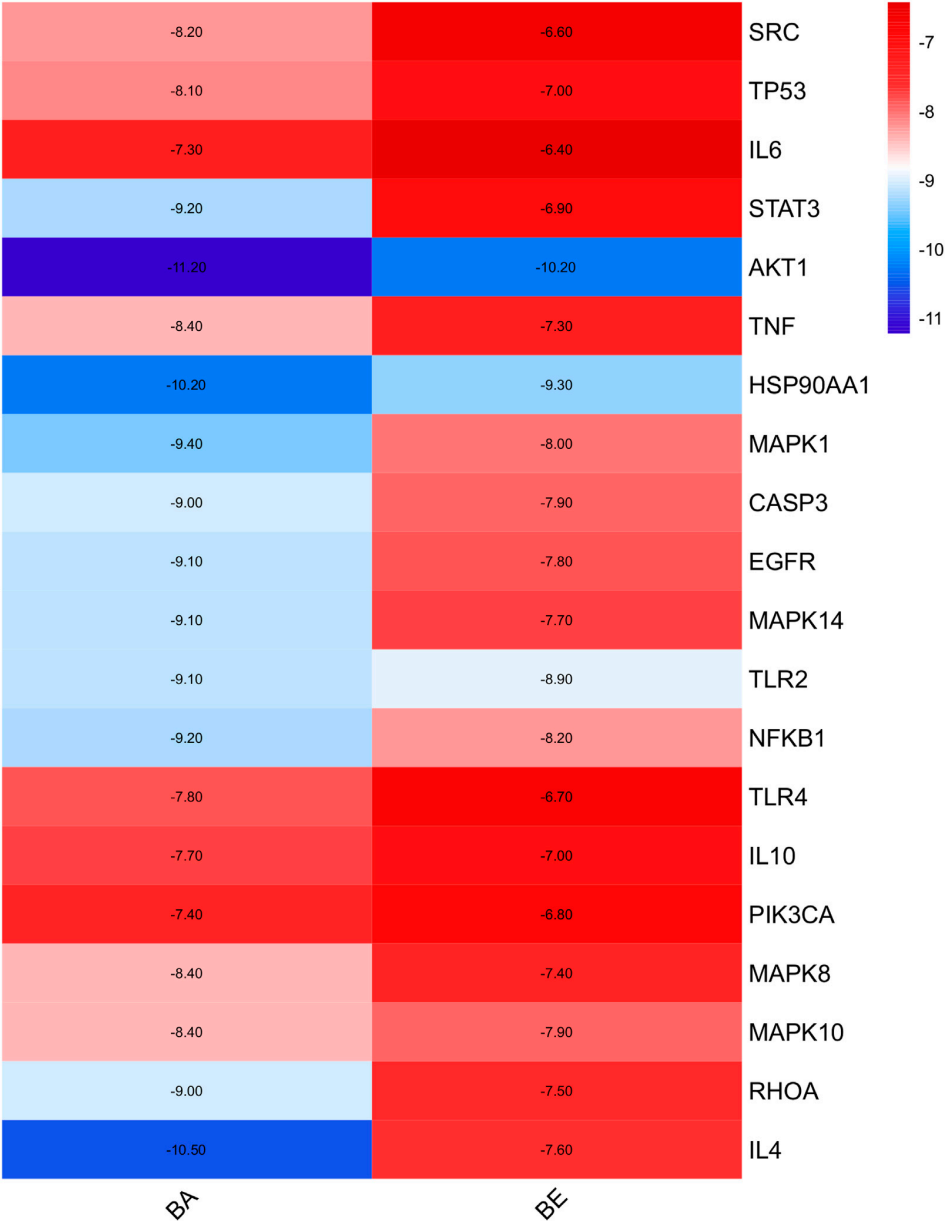
Molecular docking analysis.

Molecular docking simulations were performed to investigate the binding affinities of 20 random selected core proteins with both BA and BE, aiming to validate the results of network pharmacology. As depicted in Figure 5, the binding energies of both BA and BE with these 20 crucial proteins were all below −5 kcal/mol. Remarkably, the binding affinity to AKT1 was the most pronounced, with BA and BE exhibiting binding energies of −11.2 kcal/mol and −10.2 kcal/mol, respectively. Additionally, across the board, the binding energies of the aforementioned 20 significant proteins with BA were generally lower than those with BE, implying that BA may possess superior binding activity with the aforementioned proteins.

**FIGURE 6:**
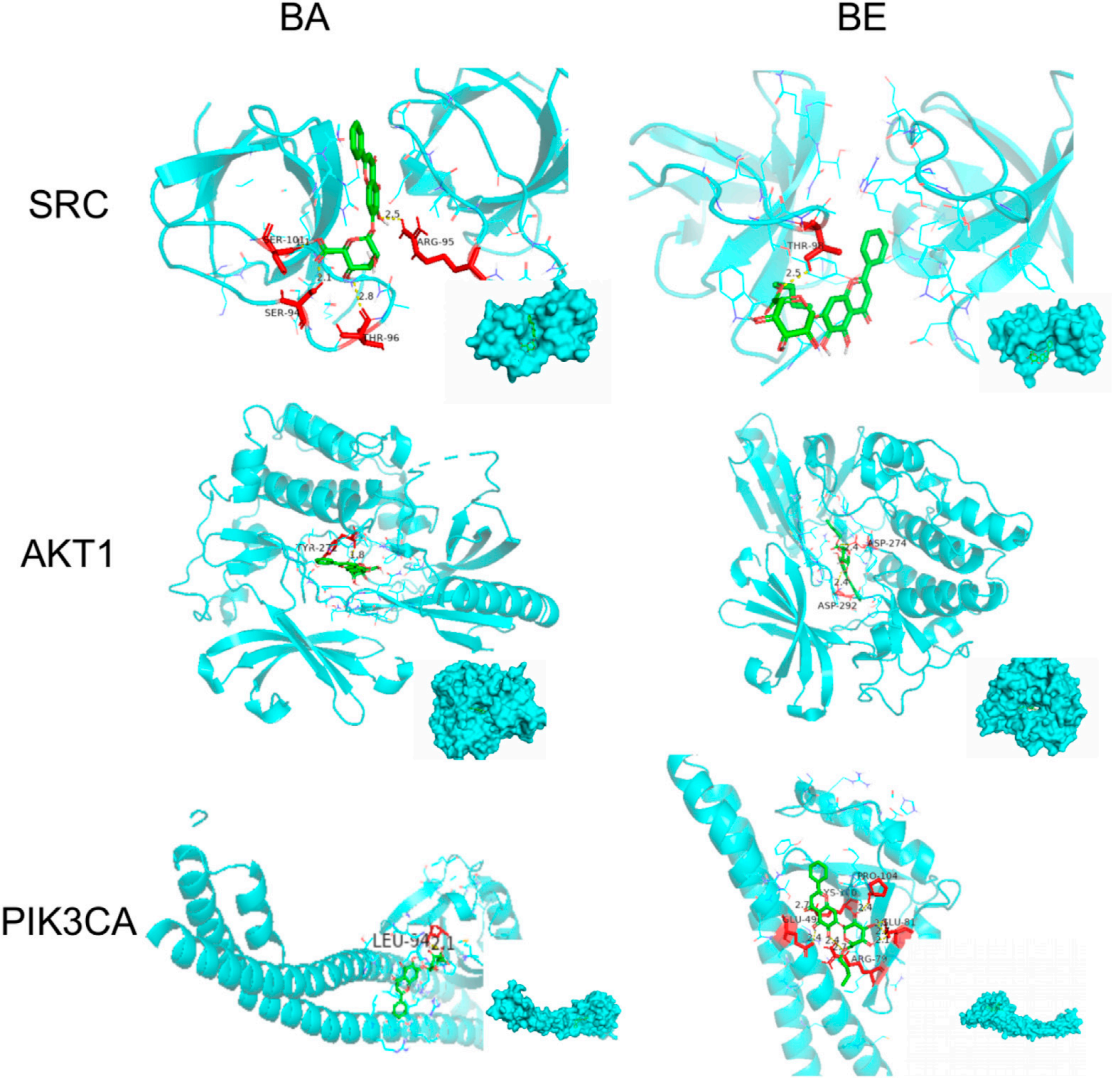
Molecular docking detail. Note: Green is the drug structure of BA or BE, blue is the protein, red is the amino acid residues interacting with the drug, and the below right corner of the figure shows the binding position of the drug to the protein.

In order to elucidate the molecular docking conformation and intricacies of the interactions between BA and BE with the target proteins, the molecular docking results of BA and BE with several key proteins are presented as an illustrative examples, and the results are presented in Figure 6 and Table 2. As shown in Figure 6, the basic structures of BA and BE were both located at similar positions of these target protein active sites. BA formed a hydrogen bond with the amino acid residue TYL-272 in the active site of AKT-1 with a distance of 1.8 Å. Conversely, BE established hydrogen bonds with the amino acid residues ASP-274 and ASP-292 in its active site, with bond distances of 2.4 Å for both (Table 2). Analogously, the interaction modes of BA and BE with PIK3CA, and SRC were depicted in Figure 6. In the active sites of PIK3CA and SRC, there was only one amino acid residue involved in hydrogen bonding interactions with BA, whereas there were 3 and 4 amino acid residues participating in interactions with BE, respectively (Table 2).

**TABLE 2:** The binding model of BA and BE with target proteins.

	BA		BE	
Protein	Amino Acid	Bond length (Å)	Amino Acid	Bond length(Å)
AKT-1	TYR-272	1.8	ASP-274	2.4
			ASP-292	2.4
PIK3CA	LEU-942	2.1	PRO-104	2.4
			GLU-290	2.4
			ARG-79	2.7
SRC	THR-98	2.5	ARG-95	2.5
			SER-101	1.1
			SER-94	2.1
			THR-96	2.8

### 3.5 Mechanism validation *in vitro*

**FIGURE 7:**
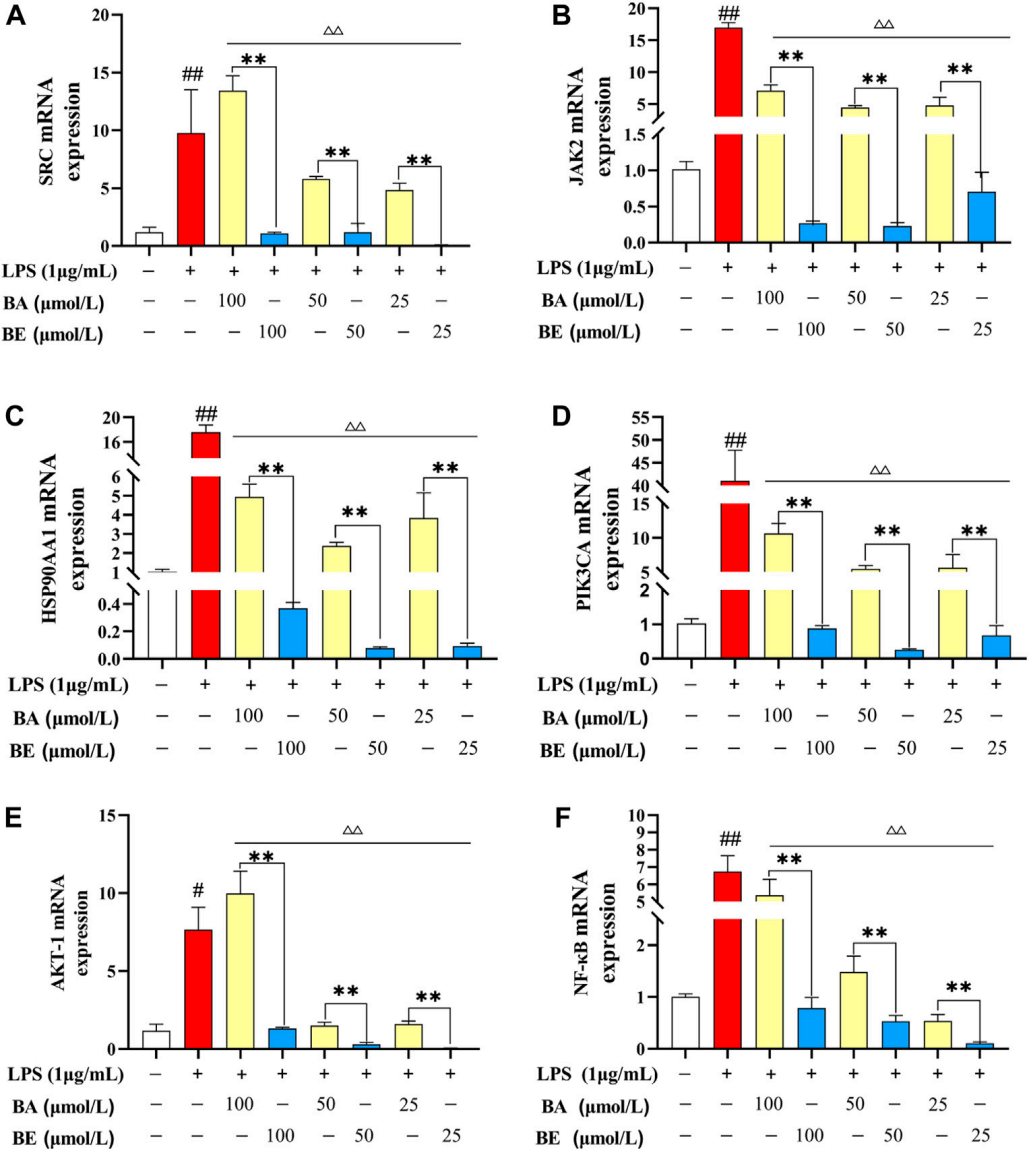
Relative mRNA expression level detection. **(A)** SRC. **(B)** JAK2. **(C)** HSP90AA1. **(D)** PIK3CA. **(E)** AKT-1. **(F)** NF-κB. Note: # P < 0.05, ## P < 0.01 compared with the CC group; ▵ P < 0.05,▵▵ P < 0.01 compared with the LPS group; * P < 0.05, ** P < 0.01 compared with BA group (N=3).

Among the top 10 signaling pathways relate to BA and BE in the context of inflammation, the PI3K-AKT signaling pathway was the common one, indicating that this pathway may severely influence the pathogenesis of inflammation. Therefore, the key targets involved in the PI3K-AKT signaling pathway, including PIK3CA, JAK2, HSP90AA1, AKT-1, NF-κB, and SRC mRNA expression levels, were examined using qRT-PCR. As depicted in Figure 7, compared to the CC group, the mRNA expression levels of SRC, JAK2, HSP90AA1, PIK3CA, AKT-1, and NF-κB in the LPS group exhibited a significant elevation (*p* < 0.01). Within the concentration range of 25–100 μmol/L, treatment with BA induced a remarkable reduction in the mRNA expression levels of JAK2, HSP90AA1, PIK3CA, and NF-κB in the model cells (*p* < 0.01). In contrast, treatment with BE resulted in a significant decrease in the mRNA expression levels of SRC, JAK2, HSP90AA1, PIK3CA, AKT-1, and NF-κB (*p* < 0.01). Notably, of particular significance, BE treatment at different concentrations exhibited a markedly superior reduction in the mRNA expression levels of SRC, JAK2, HSP90AA1, PIK3CA, AKT-1, and NF-κB in comparison to the equivalent concentrations of BA treatment (*p* < 0.01).

### 3.6 Oil-water partition and transmembrane ability detection

**FIGURE 8:**
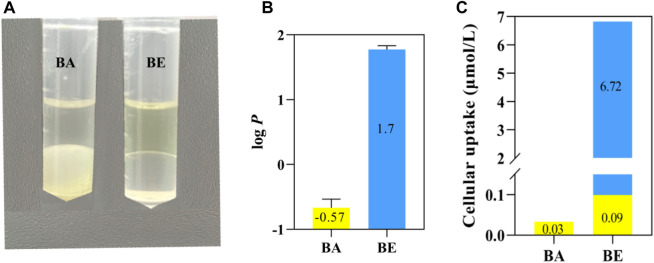
Oil-water partition and transmembrane ability detection of BA and BE. **(A)** Solubility of BA and BE in octanol and water. **(B)** log *p*-value of BA and BE. **(C)** Transmembrane ability detection of BA and BE.

The distribution and partition coefficient determination results of BA and BE in water-saturated octanol and octanol-saturated water solutions are presented in Figures 8A,B. As illustrated in Figure 8A, BA appeared as a white turbid suspension in water (lower layer) and as a light yellow transparent solution in octanol (upper layer). In contrast, BE was a yellow transparent solution in octanol (upper layer) and a colorless transparent solution in the aqueous phase (lower layer), indicating a significant alteration in the distribution of BE within the octanol-water binary solvent system. Figure 8B displays the oil-water partition coefficient determination results of BA and BE, with a log *p*-value of −0.5 for BA and a log *p*-value of 1.7 for BE.

To explore the transmembrane capabilities of BA and BE, the drug accumulation in RAW264.7 cells treated with BA and its derivatives was quantitatively determined using HPLC method. As depicted in Figure 8C, after a 4 h exposure to RAW264.7 cells, only a nominal concentration of 0.03 μmol/L BA was detected in cells treated with BA. Conversely, cells treated with BE exhibited a cumulative presence of both BA and BE, with accumulation levels of 0.09 μmol/L and 6.72 μmol/L, respectively. These findings indicate that BE exhibits superior cell membrane permeability compared to BA and suggests that BE undergoes partial hydrolysis upon cellular entry.

## 4 Discussions

Numerous studies have demonstrated the potential of BA as an effective anti-inflammatory agent for both local and systemic inflammation (Gour et al., 2021; Rizzo et al., 2021; Yan et al., 2021; Sharawi et al., 2023). Despite its promising properties, the limited bioavailability of BA hinders its widespread clinical application (Ibrahim et al., 2022; Wen et al., 2023). Therefore, this research utilized a straightforward preparation method to produce high-quality BE without the need for intricate chromatography procedures. Examination of the synthesized products revealed a yellow powder resembling BA in appearance (Figure 2A). Subsequently, various methods were utilized to further characterize its structure and purity. Full-wavelength scanning revealed a similarity in the full spectrum between the modified product and BA, suggesting the core conjugation structure of the reaction product remained unaltered (Figure 2B). FT-IR analysis showed noticeable alterations in the vibrational absorption peaks corresponding to the carboxyl group of BA due to chemical modification, along with the detection of distinct methyl and ethyl vibrational peaks, confirming the successful of the BA esterification reaction (Figure 2C). MS detection enabled precise determination of the compound’s molecular weight. In our experiment, the molecular weight of the synthesized product aligned with the anticipated weight of BE, verifying the successful synthesis of BE (Figure 2D). HPLC analysis serves as a pivotal approach for determining compound purity. The results showed that BE exhibited a prolonged retention time (106.665 min) and a well-defined peak for BE, indicating increased hydrophobicity compared to BA. Purity determination through peak area normalization confirmed a purity of 93.75% for BE (Figure 2E).

Mononuclear macrophages play a pivotal role as both effector and regulatory in inflammatory responses, orchestrating the onset and resolution of inflammation through their phagocytic capabilities and the secretion of cytokines, including IL-1β, IL-6, IL-10, and TNF-α (Xu and Li, 2018). Consequently, activated macrophages are considered the principal catalysts in the pathogenesis of a multitude of inflammatory disorders and are commonly serve as model cells in inflammatory research. LPS, a component of the outer wall of the cell wall of Gram-negative bacteria, is known to activate macrophages, prompting the production of NO and other pro-inflammatory cytokines, thereby commonly being utilized as an inducer to establish an inflammatory model (Shi and Zhao, 2022). Following the methodology outlined in reference (Hwang et al., 2016), we treated the RAW264.7 cells with 1 μg/mL LPS for 24 h to establish the *in vitro* inflammatory model. Consistent with a previous study that demonstrated BA could effectively suppress the expression of HMGB1 by promoting miR-181b, thereby inhibiting the HMGB1/TLR4/NF-κB pathway and preventing the overexpression of TNF-α, IL-6, IL-1β, COX, and iNOS (Yan et al., 2021), our research corroborates these findings by showing that BA significantly reduced the LPS-induced overexpression of NO, IL-1β, IL-6, iNOS, and COX-2 (*p* < 0.05) (Figure 3). Notably, our research further revealed that BE outperformed BA in its anti-inflammatory effects, indicating that BE exhibits enhanced bioactivity. However, what are the potential mechanisms underlying the strong anti-inflammatory effects of BE?

Network pharmacology, a burgeoning interdisciplinary field, provides novel insights into the mechanisms of drug action by leveraging bioinformatics and computational techniques to dissect the complex interplay among molecules, proteins, genes, and other biological entities (Zhao et al., 2023). In our study, it was found that there were 558 potential targets for BA, and 641 potential targets for BE through network pharmacology analysis. Concurrently, 3,252 genes were pinpointed as closely related to inflammation processes. The intersection of targets related to inflammation revealed 334 for BA and 378 for BE. Based on this, PPI networks were constructed separately for these intersection targets and underwent topological analysis. It was found that there were 59 core anti-inflammatory targets for BA and 66 for BE with degree values exceeding 10 (Figures 4A–F). Subsequently, KEGG pathway enrichment analysis of these core targets revealed that the PI3K-AKT pathway is a common enriched pathway in the anti-inflammatory mechanisms of both drugs (Figures 4G,H), a finding that is congruent with the previous research that have highlighted the alleviating effect of BA on hyperuricemic nephropathy (Liu et al., 2023).

Molecular docking, a computational simulation technique, predicts the binding affinity of small molecules to target proteins by calculating binding energies (Fu et al., 2022). Generally, lower binding energies between ligands and receptors indicate greater stability of the conformation and higher binding affinity, which correlates with stronger pharmacological effects (Halder et al., 2024). Specifically, binding energies below 0 kcal/mol suggest spontaneous ligand-receptor binding, while values more negative than −4 kcal/mol indicate some degree of docking activity, with thresholds of −5.0 and −7.0 kcal/mol signifying good and strong docking activity, respectively (Jiang et al., 2022). In our study, we performed molecular docking on 20 core targets common to both BA and BE, revealing that their binding energies were less than −5.0 kJ/mol (Figure 5), indicative of good binding activity. Notably, AKT1 exhibited the lowest binding energies with both BA and BE, at −11.2 kJ/mol and −10.2 kJ/mol, respectively, indicating its pivotal role in the anti-inflammatory mechanism. These docking results, in conjunction with KEGG pathway enrichment analysis from network pharmacology, led us to hypothesize that the anti-inflammatory effects of BA and BE are likely mediated through the PI3K-AKT signaling pathway.

The PI3K-AKT pathway plays a vital role in regulating the inflammatory response, with PI3K and AKT being the most critical proteins within this pathway (Meng et al., 2021). NF-κB, another crucial participant in the inflammatory response, is activated by various extracellular and intracellular stimui and functions as a downstream effector of PI3K-AKT (Xie et al., 2021). Specifically, during inflammation, NF-κB is activated by AKT, initiating the degradation of IκBα and thereby triggering the NF-κB pathway cascade (Li et al., 2023). Existing study has shown that BA can significantly alleviate ulcerative colitis induced by 2,4,6-trinitrobenzene sulfonic acid (TNBS) by blocking the PI3K-AKT signaling pathway, thereby reducing the release of IL-6, TNF-α, and IL-1β (Zhu et al., 2020). To corroborate the findings from network pharmacology and molecular docking, we utilized LPS-induced RAW264.7 cells to establish an inflammatory model and explored the impact of BA and BE on the expression levels of core targets enriched in the PI3K-AKT pathway. The results showed that both BA and BE could effectively counteract the upregulation of SRC, JAK2, HSP90AA1, PIK3CA, AKT-1, and NF-κB in the model cells (Figure 7). Notably, BE exhibited a superior effect than BA, indicating its enhanced capacity to inhibit the PI3K-AKT signaling pathway and elicit a robust anti-inflammatory response, thereby validating the network pharmacology predictions.

Interestingly, molecular docking studies revealed that BA exhibits lower binding energies with the core targets compared to BE (Figure 5), which paradoxically suggests that individual BE molecule might have weaker binding activity. This results seem to contradict our earlier findings showing that BE has superior inhibitory effects on inflammatory factor release, indicating a more potent anti-inflammatory activity than BA. To reconcile this discrepancy, we measured the oil-water partition coefficients and transmembrane permeability of BA and BE. The Log *p* values often used to reflect the distribution of compounds in the oil-water phases (Lin et al., 2021). A higher value indicates greater lipophilicity of the compound, and when the log *p*-value falls within the range of 1–3, it suggests optimal absorption efficiency of the compound (Sarma et al., 2021). The present study found that the Log *p*-value of BE is significantly higher than that of BA, at 1.7 (Figure 8B), indicating that BE possesses better lipid solubility and stronger absorption capability compared to BA. This suggests that BE may achieve higher cellular concentrations than BA, potentially explaining its stronger anti-inflammatory activity. To test this hypothesis, we quantified the cellular accumulation of BA and BE in RAW264.7 cells using HPLC method. The results showed that BE accumulates in cells at levels over 200 times higher than BA. Furthermore, BA was also detected in cells treated with BE (Figure 8C), suggesting that BE undergoes partial hydrolysis to BA upon cellular entry. Collectively, these results support the hypothesis that BE’s higher cellular load contributes to its enhanced anti-inflammatory activity compared to BA.

## 5 Conclusion

In summary, we successfully synthesized BE and, for the first time, demonstrated its enhanced anti-inflammatory activity in comparison to BA. Utilizing a multi-faceted approach encompassing network pharmacology, molecular docking, and *in vitro* experiments, we substantiated that BE potentially exert its anti-inflammatory activity through the PI3K-AKT signaling pathway. Further research revealed that while the anti-inflammatory activity of the individual BE molecule might be weaker than that of BA, its enhanced transmembrane ability and higher cellular load are pivotal factors that contribute to its overall more efficacious anti-inflammatory profile. These findings underscore the importance of BE’s pharmacokinetic properties in its therapeutic potential.

## Data Availability

The original contributions presented in the study are included in the article/Supplementary Material, further inquiries can be directed to the corresponding authors.
